# Technological evaluation of strategies to get out of bed by people with Parkinson's disease: Insights from multisite wearable sensors

**DOI:** 10.3389/fmedt.2022.922218

**Published:** 2022-08-25

**Authors:** Jirada Sringean, Chusak Thanawattano, Roongroj Bhidayasiri

**Affiliations:** ^1^Chulalongkorn Centre of Excellence for Parkinson's Disease & Related Disorders, Department of Medicine, Faculty of Medicine, Chulalongkorn University and King Chulalongkorn Memorial Hospital, Thai Red Cross Society, Bangkok, Thailand; ^2^National Science and Technology Development Agency (NSTDA), Pathumthani, Thailand; ^3^The Academy of Science, The Royal Society of Thailand, Bangkok, Thailand

**Keywords:** Parkinson's disease, getting out of bed, early morning akinesia, nocturia, wearable sensor, objective outcome measure

## Abstract

**Background:**

Difficulty getting out of bed is a common night-time and early morning manifestation of Parkinson's disease (PD), rated by 40% of the patients as their most concerning motor symptoms. However, current assessment methods are based on clinical interviews, video analysis, and clinical scales as objective outcome measures are not yet available.

**Objective:**

To study the technical feasibility of multisite wearable sensors in the assessment of the supine-to-stand (STS) task as a determinant of the ability to get out of bed in patients with PD and age-matched control subjects, and develop relevant objective outcome measures.

**Methods:**

The STS task was assessed in 32 patients with PD (mean Hoehn and Yahr; HY = 2.5) in the early morning before their first dopaminergic medication, and in 14 control subjects, using multisite wearable sensors (NIGHT-Recorder®; trunk, both wrists, and both ankles) in a sleep laboratory. Objective getting out of bed parameters included duration, onset, velocity and acceleration of truncal rotation, and angle deviation (a°) from the *z*-axis when subjects rose from the bed at different angles from the *x*-axis (10°, 15°, 30°, 45°, and 60°) as measures of truncal lateral flexion. Movement patterns were identified from the first body part or parts that moved. Correlation analysis was performed between these objective outcomes and standard clinical rating scales.

**Results:**

Compared to control subjects, the duration of STS was significantly longer in patients with PD (*p* = 0.012), which is associated with a significantly slower velocity of truncal rotation (*p* = 0.003). Moderate and significant correlations were observed between the mean STS duration and age, and the Nocturnal Hypokinesia Questionnaire. The velocity of truncal rotation negatively and significantly correlated with HY staging. Any arm and leg moved together as the first movement significantly correlated with UPDRS-Axial and item #28. Several other correlations were also observed.

**Conclusion:**

Our study was able to demonstrate the technical feasibility of using multisite wearable sensors to quantitatively assess early objective outcome measures of the ability of patients with PD to get out of bed, which significantly correlated with axial severity scores, suggesting that axial impairment could be a contributing factor in difficulty getting out of bed. Future studies are needed to refine these outcome measures for use in therapeutic trials related to nocturia or early morning akinesia in PD.

## Introduction

Nocturnal hypokinesia is a common manifestation of Parkinson's disease (PD), affecting up to 70% of the patients and becoming symptomatic from the moderate stage of the disease (1). While early literature refers to nocturnal hypokinesia as difficulty changing position in bed, recent studies have identified that there are two main clinical manifestations of nocturnal hypokinesia, consisting of impaired ability to turn in and difficulty getting out of bed. These two features have recently been combined to form an operational definition of nocturnal hypokinesia as a decreased ability to perform sufficient axial rotation and/or trunk flexion to turn in or get out of bed as a result of axial and limb muscle incoordination (1–3). These impairments may occur in isolation when patients toss and turn in bed during sleep, or as a continuum when they attempt to turn in and get out of bed due to nocturia during the night or in the early morning (4, 5).

In clinical practice, nocturnal hypokinesia, assessed by clinical interviews and screening instruments, identified that 65–70% of the patients with PD are affected overall, and 39% of the patients rated this as their most troublesome night-time symptom (6). Amongst the scales recommended by the Movement Disorder Society Task Force, only the modified Parkinson's Disease Sleep Scale (PDSS-2) has a specific item to rate the overall severity of nocturnal immobility (#9: Did you feel uncomfortable at night because you were unable to turn around in bed or move due to immobility?), but no specific items that ask patients about their ability to get out of bed (7, 8). More recently, the Nocturnal Hypokinesia Questionnaire (NHQ) has been developed and validated with 10 items to be completed independently by patients and their carers with two specific items (item #3 Do you have any difficulty getting out of bed so you need several attempts before you succeed?; item #4 Do you require assistance so you ask for help or hold onto bed rails to get out of bed?) dedicated to evaluating a patient's difficulty in getting out of bed (5, 9). When NHQ was administered to moderate-stage patients with PD (mean HY = 2.6), difficulty in getting out of bed was identified in 57.9% (item #3) and 43.4% (item #4) of the patients, respectively, with almost a comparable number of patients affected by impaired turning in bed (55.3–61.8%) (9). In a separate study in Korean patients with PD, item #3, related to difficulty in getting out of bed, was rated as the most common symptom (62%) within the NHQ, followed by item #2, related to lying in a supine position for most of the night (10).

With advances in circuit technology, wearable sensors have been developed to study night-time movement patterns in patients with PD. When multisite inertial sensors were applied to both wrists, ankles, and trunk of mild-to-moderate patients with PD and their spouses to monitor night-time movements in their own bedroom environment, patients with PD had significantly fewer incidents of rolling over, turned with a smaller degree, less velocity, and acceleration when compared to their spouses, suggesting that difficulty turning in bed was primarily related to impaired axial rotation (5). Moreover, impaired axial rotation worsened as the night progressed (11). A subsequent study with one sensor on the lower back also confirmed similar findings, demonstrating reduced nocturnal movements that were associated with increased motor severity, worsened dysautonomia, cognition, and higher dosages of dopaminergic medications (4). While wearable sensors have been used in the study of turning in bed in PD, to the best of our knowledge, this approach has not been applied in the assessment of getting out of bed, and very few studies provided descriptive information based on video-based analysis on how patients with PD got out of their beds. When movements were categorized into three body regions (head and trunk, arm, and legs), there were a variety of movement patterns that patients with PD utilized for getting out of bed with “come to sit,” “multi-push and double-push,” and “synchronous” being the most common strategies for head and trunk, arms, and legs, respectively (12). A more recent video analysis study yielded similar findings, although the “step off” of the legs strategy was also utilized as frequently as the “synchronous” strategy (13). When compared to movement patterns used by healthy elderly individuals, patients with PD employed fewer “roll off” strategies, which may reflect impaired axial rotation as observed in our sensor-based studies on turning in bed (5, 12, 14).

Expanding the understanding of patients' difficulties in getting out of bed has several important clinical implications. In addition to a decreased ability to turn in bed, difficulty getting out of bed represents another clinical dimension that many patients with PD encounter when going out to the toilet during the night or getting up to start the day in the early morning. While the evidence is clear that nocturnal hypokinesia, when considered as a whole, negatively affects sleep quality, sleep efficiency, and quality of life of patients with PD (15, 16), difficulty in getting out of bed, as an isolated symptom, was rated as an emerging disability amongst patients with PD after a 2-year subsequent follow-up, and was the most frequent activity of daily living (ADL) to be assisted by carers of patients with PD (17, 18). Similarly, another separate study identified the problem of getting out of bed as a factor associated with the presence of a carer, and it was rated by 39.6% of the patients with PD as their commonly concerning motor symptoms (19). When getting out of bed is considered as a spectrum of early morning off (EMO), it was found to be very common, affecting 72.4% of patients with PD, with bradykinesia or rigidity and fatigue or sleepiness the most common motor and non-motor symptoms, respectively (20). Moreover, difficulties getting out of bed were also experienced by patients with PD whilst waiting for their first dose of levodopa to work (21). Although video recording is a good instrument to provide visual analysis that assists clinicians in the characterization of motor phenotypes of getting out of bed, a sensor-based evaluation has the advantage of obtaining precise, accurate, and quantitative data that is related to the kinematics of getting out of bed. Therefore, the aim of this study is to objectively evaluate with multisite wearable sensors the ability and strategies used by patients with PD when getting out of bed and compare them with age-matched healthy controls.

## Patients and method

### Participants

Participants of this study were patients at the Chulalongkorn Center of Excellence for Parkinson's Disease and Related Disorders (ChulaPD, www.chulapd.org) with the diagnosis of PD according to the United Kingdom Parkinson's Disease Society Brain Bank criteria. Control subjects were without known neurological disorders and were matched to the patients with PD with respect to age. We excluded subjects if they were bedridden, had a history of other neurological and musculoskeletal disorders, including low back pain that may compromise their ability to get out of bed, and took any hypnotic or sedative drugs. Subjects with a history of cerebrovascular disorders or focal neurological signs suspected of previous cerebrovascular events were also excluded. All participants were carefully examined by two independent movement disorder neurologists (JS and RB) to ensure that there were no clinical features as stated in the exclusion criteria. Clinical demographics and rating scales, including Hoehn and Yahr (HY) stage and Unified Parkinson's Disease Rating Scale (UPDRS), were evaluated in all the patients with PD. The UPDRS axial score was calculated as the summation of items 18, 22, 27, 28, 29, and 30 of the UPDRS section 3 (11). The NHQ was administered to all PD subjects with 10 items divided into four domains, including turning in bed, getting out of bed, parkinsonian motor symptoms, and others (9). Levodopa Equivalent Daily Dose (LEDD) was determined using the standardized protocol (22). A specific night-time LEDD that was the combination of the last dose of dopaminergic medication before bedtime and a rotigotine transdermal patch was also calculated. All scales were rated by two independent movement disorder neurologists (JS and RB) who had to agree on their rating assessments. In case of a discrepancy, both raters assessed the evidence once again and arrived at a consensus. The study was approved by the Human Ethics Committee of the Faculty of Medicine, Chulalongkorn University (IRB number 153/57). All subjects gave written informed consent before the enrolment of the study in accordance with the declaration of Helsinki.

### Wearable sensors

**Figure 1:**
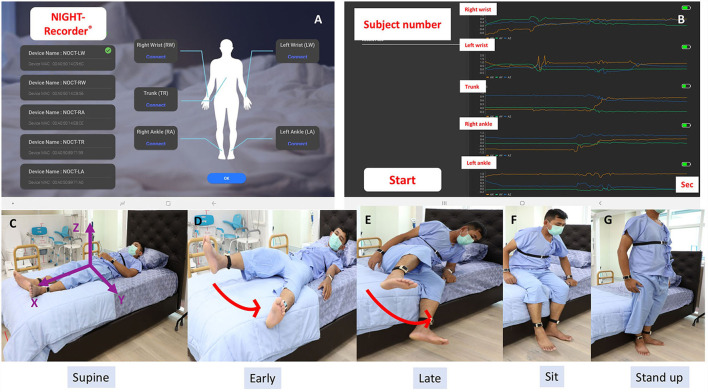
**(A)** The NIGHT-Recorder® application interface demonstrated a successful connection with a set of five wearable sensors. **(B)** Examples of sensor signals from the NIGHT-Recorder® during the supine-to-stand .task (orange: *x*-axis; green: *y*-axis; blue: *z*-axis). **(C)** Three axes (*x, y*, and *z*) orientation of the trunk sensor. **(D–G)** Video recording of a control subject demonstrated “leg first” as the first movement pattern of a supine-to-stand task.

The inertial sensor system (the NIGHT-Recorder®) used in this study was developed by our group with technical development and experimental verification described elsewhere (23). It consists of a 16-bit digital output triaxial integrated microelectromechanical system (iMEMS) accelerometer and gyroscope that are capable of measuring linear and angular accelerations in three translational planes (*x, y*, and z) on the patient with an axial sensor, as shown in Figure 1. The recordings were obtained using a 20-Hz sampling rate with a low pass filtering at 12 Hz. The NIGHT-Recorder® system consists of five wearable sensors attached to both wrists, both ankles, and the trunk, paired *via* Bluetooth to the NIGHT-Recorder application operating on an Android tablet (Version 9.0) (Figure 1).

### Procedure

The task of getting out of bed was assessed by the supine-to-stand (STS) task, which is considered a measure of basic functional ability, and a useful tool to examine functional motor competence and a general health status marker (24, 25). The study took place in the sleep laboratory of ChulaPD where all subjects wore the NIGHT-Recorder® on both wrists and ankles, and on their trunk at the subxiphoid level above their clothes, fastened with a Velcro band. The task was performed on a standard queen-size bed, measuring 80 inches long and 60 inches wide, with a 3-inch firm padded surface, covered with a cotton sheet, with a floor-to-plinth surface height of 22 inches. We consider this height to be optimal as, when the subject sat on the plinth, their feet were flat on the floor and their knees at a 90° bend. A standard hospital pillow was used for head support and all subjects were asked to lie in the center of the bed. The STS task was assessed in the early morning in all subjects. In the case of patients with PD, the task was performed prior to the first dose administration of dopaminergic medications, corresponding to their OFF periods. The following instructions were given to each subject before each trial: “When the researcher says “Go,” please get out of bed the way you normally do at home and stand.” All subjects performed the task twice, one on each side of the bed, beginning on the left. The mean from both trials was used for statistical comparison. Our researchers guarded the subjects throughout the task to reduce the risk of falling by standing on the side to which the subject was getting up, near the head of the bed as the subject rose to a sitting position, so they were close enough to catch the subject if he or she were to lose their balance. Between trials, all subjects were permitted to rest for a few minutes until they felt ready to repeat the test. Following completion, patients with PD were instructed to resume their first dose of medications. All trial sessions were videotaped for the purpose of verification with sensor data.

**Figure 2:**
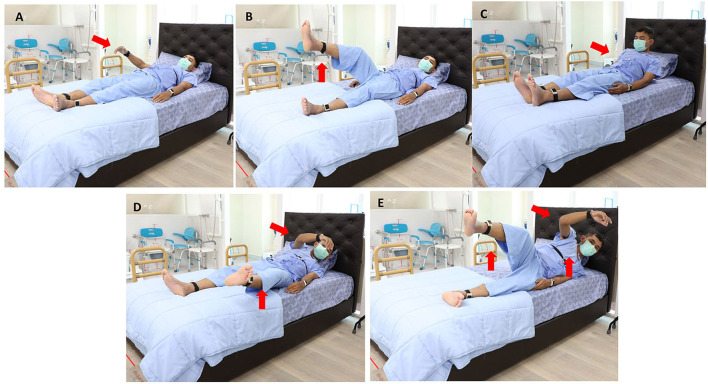
Movement patterns of the supine-to-stand task were categorized according to the first body part or parts that moved. **(A)** Arm first; **(B)** leg first; **(C)** trunk first; **(D)** any arm and leg first together; **(E)** trunk with any arm and/or leg first.

**Figure 3:**
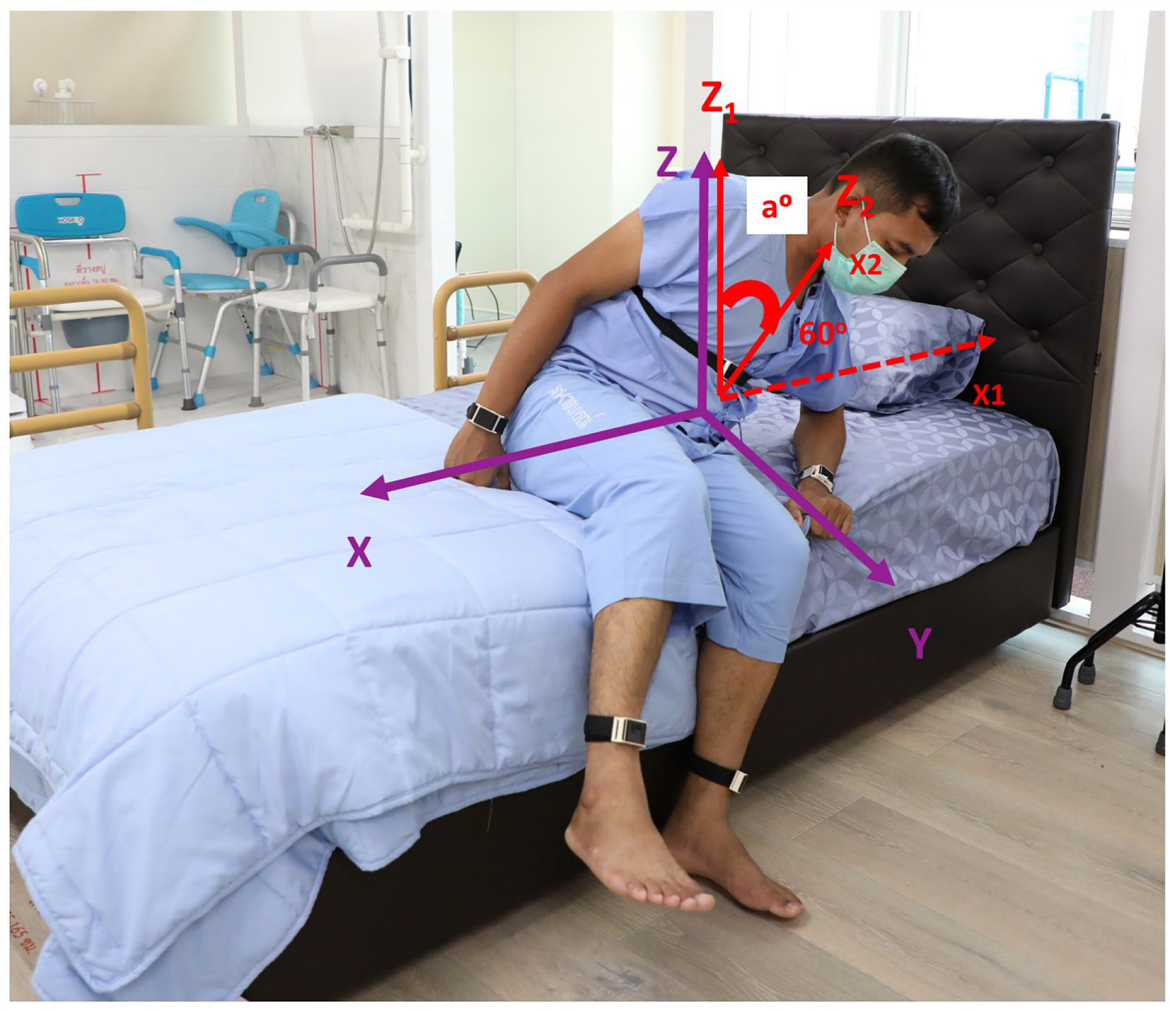
An attempt to perform a supine-to-stand task in a control subject with superimposed three axes orientation of the sensor on the trunk (purple) and angle deviation (a°) as shown when a subject rose from the bed at 60° from the *x*-axis.

The ability to perform the STS task was evaluated if subjects were able to complete the task from the initial movement that occurred following the “Go” prompt to a complete stand on two feet by the bedside. The cut point of definition of limb movement was defined as a change of at least 15° from the previous position, but not necessarily sustained (5). Objective characteristics of getting out of bed consisted of the following: (1) STS duration, the interval between the indicator marked on the tablet application as the “Go” instruction was given until subjects were in a full standing position. This outcome has been shown to have sufficient variability to distinguish individuals of all ages, without having ceiling or floor effects (24); (2) onset duration to initiate the task, defined by the interval between the “Go” indicator and the first body part or parts being moved; and (3) the first body part or parts that moved to initiate the STS task, categorized into arm first, leg first, trunk first, any arm and leg first together, and trunk with any arm and/or leg first, expressed as a percentage (Figure 2). Truncal rotation and lateral flexion were chosen as the two outcomes to determine flexibility having recently been shown as valid measures of axial rigidity (26). Velocity and acceleration were included as measures of truncal rotation, and the angle deviation (a°) from the *z*-axis when subjects rose from the bed at different angles from the *x*-axis (10°, 15°, 30°, 45°, and 60°) were included as measures of truncal lateral flexion (Figure 3). Signal processing was performed using a forward derivative method on the accelerometer data to obtain its derivatives on the Sleep Motion Analyser Software (Version 2.0) running on Python (Version 3.5.0).

### Statistical analysis

Baseline characteristics of all subjects were summarized using either means, standard deviation, or frequencies and percentages as appropriate. The normal distribution of STS parameters was performed with the Kolmogorov Smirnov test. Unpaired student *t*-tests were used to compare getting out of bed parameters between the patients with PD and age-matched healthy subjects. The Chi-square test was used for comparing categorical parameters. Pearson's correlation coefficient was calculated to determine correlations between getting out of bed parameters and demographic data and scale-based assessments. A *p*-value of <0.05 was considered statistically significant. Statistical analysis was performed using the SPSS software (Version 22.0, SPSS Inc., Chicago IL.).

## Results

Thirty-two patients with PD (15 M, 17 F; mean age 63.1 ± 10.7 years) and 14 age-matched control subjects (5 M, 9 F; mean age 61.1 ± 9.1 years) participated in the study. Three patients with PD were excluded as they were unable to complete the STS task, resulting in a final enrolment of 29 patients with PD (14 M, 15 F; mean age 62.9 ± 10.5 years). Demographic data and disease characteristics of all subjects are shown in Table 1. There were no significant differences between age, weight, or waist circumference between the two groups. The majority of the patients with PD were in the moderate disease stage, as reflected by a mean HY staging of 2.5 (SD = 0.6) and a mean disease duration of 11.1 years (SD = 5.5). History of motor fluctuation was determined from the clinical history and the presence of nocturnal hypokinesia was determined by NHQ in every PD patient. While all 29 PD subjects were able to complete the STS task without any assistance, they expressed some degree of difficulty in performing this task, perceiving their bodies as being heavy to roll, or their back being very stiff to get up. STS duration was significantly longer in patients with PD than in control subjects (9.81 ± 5.40 vs. 5.87 ± 1.63 s, *p* = 0.012), but there was no significant difference between the onset of the STS task between the two groups (Table 2). In addition, there was no significant difference in STS parameters between the male and female gender. While both groups utilized trunk with any arm and leg together as the most frequent first movement in the STS task, patients with PD used this movement pattern significantly less than control subjects (44.8 vs. 78.6%, *p* = 0.005). In contrast, patients with PD used any arm and leg as the first movement of the STS task slightly more frequently than control subjects (13.8 vs. 0%, *p* = 0.046) (Table 2). There was a tendency in the patients with PD to use the arm, leg, or trunk as an isolated first movement more frequently than in control subjects although the number of movements was not statistically different between the two groups. Truncal rotation velocity was significantly slower in patients with PD than control subjects (11.06 ± 6.50 vs. 16.46 ± 5.17 cm/s, *p* = 0.003) although the comparison of acceleration was not statistically significant. The a° was not significantly different between the two groups, but there was a trend toward larger deviation in control subjects as compared to the patients with PD when they rose from the bed at 10°, 30°, 45°, and 60°. None of the subjects experienced falls or any other adverse events during the trials.

**Table 1:** Clinical demographics of patients with Parkinson's disease and control subjects.

**Clinical characteristic**	**Parkinson's disease patients (*n* = 29)**	**Control subjects** ** (*n* = 14)**	***p* value**
Age (years)	62.9 ± 10.5	61.1 ± 9.1	0.556^¶^
Gender	M: 14; F: 15	M: 5; F: 9	0.523^†^
Weight (kg)	56.5 ± 14.4	62.8 ± 12.4	0.145^¶^
Height (cm)	161.4 ± 11.1	157.9 ± 8.6	0.257^¶^
Waist circumference (cm)	81.4 ± 10.9	88.1 ± 9.3	0.053^¶^
Age of onset (years)	51.4 ± 12.0	-	
Disease duration (years)	11.1 ± 5.5	-	
Hoehn& Yahr staging	2.5 ± 0.6	-	
• HY 1.0	0/29 (0%)	-	
• HY 1.5	2/29 (6.9%)	-	
• HY 2.0	8/29 (27.6%)	-	
• HY 2.5	9/29 (31.0%)	-	
• HY 3	8/29 (27.6%)	-	
• HY 4	2/29 (6.9%)	-	
• HY 5	0/29 (0%)	-	
UPDRS III	28.7 ± 11.3	-	
UPDRS axial scores	7.3 ± 3.3	-	
UPDRS item 28 (posture)	1.1 ± 0.7		
Total LEDD (mg/day)	775.0 ± 480.6	-	
Nighttime LEDD (mg/day)	111.7 ± 109.4	-	
Total NHQ	2.0 ± 2.7	-	

UPDRS, Unified Parkinson's Disease Rating Scale; LEDD, Levodopa Equivalent Daily Dose; NHQ, Nocturnal Hypokinesia Questionnaire.

Statistical significance (*) is defined by p ≤ 0.05.

¶ Unpaired student t-test.

† Chi-square test.

**Table 2:** Comparison of getting out of bed parameters between patients with Parkinson's disease and control subjects.

**Getting out of bed parameter**	**Parkinson's disease patients (*n* = 29)**	**Control subjects** **(*n =* 14)**	***p* value^¶^**
Duration (s)	9.81 ± 5.40	5.87 ± 1.63	0.012*
Onset (s)	0.33 ± 0.29	0.27 ± 0.40	0.640
**First body part, or parts, that moved (%)**	**Parkinson's disease patients**	**Control subjects**	***p*** **value**^†^
Any arm first (%)	10/58 (17.2%)	4/28 (14.3%)	0.760
Any leg first (%)	4/58 (6.9%)	0/28 (0%)	0.294
Trunk first (%)	8/58 (13.8%)	2/28 (7.1%)	0.482
Any arm and leg together first (%)	8/58 (13.8%)	0/28 (0%)	0.046*
Trunk with any arm and/or leg (%)	26/58 (44.8%)	22/28 (78.6%)	0.005*
**Truncal rotation**	**Parkinson's disease patients**	**Control subjects**	***p*** **value**^¶^
Velocity (cm/s)	11.06 ± 6.50	16.46 ± 5.17	0.003*
Acceleration (cm/s^2^)	2.35 ± 4.67	3.29 ± 2.22	0.365
**Truncal lateral flexion**	**Parkinson's disease patients**	**Control subjects**	***p*** **value**^¶^
a° when rising at 10° (degree)	21.93 ± 15.08	28.37 ± 14.82	0.201
a° when rising at 15°	29.14 ± 18.16	29.28 ± 15.18	0.979
a° when rising at 30°	38.10 ± 18.44	40.70 ± 24.70	0.732
a° when rising at 45°	43.01 ± 18.28	49.00 ± 19.57	0.352
a° when rising at 60°	41.37 ± 19.44	46.37 ± 23.10	0.496

Statistical significance (*) is defined by p ≤ 0.05.

¶ Unpaired student t-test.

† Chi-square test.

Angle deviation (a°) was measured from the z-axis when subjects rose from the bed at different angles from the x-axis at 10°, 15°, 30°, 45°, and 60°.

Correlation analysis was performed between STS duration and clinical demographics and scale-based assessments (Table 3). There were significant and moderate correlations between STS duration and age (*r* = 0.564, *p* = 0.002), age at onset (*r* = 0.393, *p* = 0.042), HY staging (*r* = 0.539, *p* = 0.004), UPDRS axial score (*r* = 0.585, *p* = 0.001), UPDRS item# 28 on posture (*r* = 0.444, *p* = 0.02), and NHQ total score (r = 0.411, *p* = 0.033). After using multiple linear regression analysis, the two factors that correlated with the mean duration of STS were age (beta = 0.576, *p* = 0.014) and total NHQ (beta = 0.465, *p* = 0.037). A negative moderate and significant correlation was identified between the velocity of truncal rotation and HY staging (*r* = −0.415, *p* = 0.018). In terms of first movement patterns, there were moderate and significant correlations between any arm and leg moved together first and UPDRS axial score (*r* = 0.475, *p* = 0.012) and UPDRS item #28 (r = 0.576, *p* = 0.002). Other correlations are provided in Supplementary Data 1.

**Table 3:** Correlation analysis between getting out of bed parameters and clinical characteristics of patients with Parkinson's disease.

**Clinical characteristics**	**Getting out of bed parameters**		
	**Duration r (*p* value)**	**Truncal rotation velocity** **r (*p* value)**	**Angle deviation (a°) when rising at 60°r (*p* value)**
Age	0.564 (0.002*)	−0.268 (0.176)	−0.201 (0.316)
Body weight—	−0.284 (0.151)	0.271 (0.172)	0.062 (0.759)
Waist circumference	−0.235 (0.238)	0.291 (0.141)	0.118 (0.556)
Age of onset	0.393 (0.042*)	−0.090 (0.654)	−0.066 (0.743)
Disease duration	0.192 (0.338)	−0.277 (0.162)	−0.190 (0.342)
HY	0.539 (0.004*)	−0.415 (0.018*)	−0.116 (0.563)
UPDRS-III	0.342 (0.081)	−0.282 (0.154)	−0.011 (0.958)
UPDRS-axial score	0.585 (0.001*)	−0.315 (0.109)	−0.219 (0.272)
UPDRS item# 28 (posture)	0.444 (0.020*)	−0.315 (0.110)	−0.264 (0.184)
Total LEDD	0.164 (0.415)	0.020 (0.922)	−0.033 (0.871)
Nighttime LEDD	0.257 (0.196)	−0.285 (0.150)	−0.155 (0.439)
Total NHQ	0.411 (0.033*)	−0.206 (0.302)	−0.196 (0.328)

HY, Hoehn & Yahr staging; UPDRS, Unified Parkinson's Disease Rating Scale; LEDD, Levodopa Equivalent Daily Dose; NHQ, Nocturnal Hypokinesia Questionnaire.

Pearson's correlation coefficient, Statistical significance (*) is defined when p ≤ 0.05.

## Discussion

Our sensor-based study has yielded objective outcomes of the STS task in different aspects and firstly provides a technical verification that the STS task is a valid measure of getting out of bed in PD and that it is technically feasible to apply sensors in its assessment in patients with PD in a controlled environment. Second, this study includes kinematic parameters of the STS task, demonstrating a significantly longer duration and slower truncal rotational velocity in patients with PD compared to control subjects when they attempted to get out of bed in the early morning. Our objective evidence is consistent with the previous video-based analysis that patients with PD took a significantly longer time to get out of bed compared to age-matched controls (12). Despite different techniques, the mean duration of getting out of bed from our study with a sensor-based measurement was comparable to a previously published study with a video-based analysis for both patients with PD (mean: 8.2–9.81 s) and control subjects (mean: 5.3–5.87 s), supporting the validity of both techniques (12). Whether these mean values can be used as references for this task in patients with PD and age-matched controls should be confirmed in future studies with larger sample sizes. Indeed, a decreased ability of patients with PD to get out of bed in this study was expressed in terms of long duration, the slow velocity of truncal rotation (speed: bradykinesia), but not the angle of lateral flexion (amplitude: hypokinesia), reflecting bradykinesia as a manifestation of an “OFF” period in PD (27, 28). Therefore, our findings expand the spectrum of nocturnal hypokinesia that it not only occurs during the night and worsens as the night progresses, but also extends to early morning, at least until the first dose of dopaminergic medications takes effect, when patients with PD are slower and take longer to get out of bed compared to healthy elderly populations. Significant correlations between both duration and velocity of the STS task and disease severity, as shown by HY staging, further support previous findings that nocturnal hypokinesia is usually subtle in the early stage and becomes symptomatic in moderate-stage patients with PD and beyond (1, 5, 29). Additional correlations that were significant between the duration of the STS task and the severity of axial symptoms as determined by UPDRS axial scores further support the early view that getting out of bed is primarily an axial manifestation (1).

The unique feature of our study is that we utilized multisite wearable sensors in the assessment of getting out of bed, providing important information on how limbs and trunk were coordinated to achieve the task and allowing analysis to see if the patterns in PD were similar or different from control subjects. As getting out of bed is conceptualized as a complex sequential motor skill that requires axial and limb muscle coordination to perform sufficient axial rotation and/or trunk flexion (1, 30), it is ideal to use this set of sensors to identify if there are specific movement patterns that are quantifiable from the beginning to completion of the STS task and to determine if there are distinct patterns of getting out of bed that are employed by patients with PD. However, this approach was found not to be useful as there were no common patterns that are shared by either patients with PD or control subjects when processing sensor signals from five different body locations (two wrists, two ankles, and one trunk) (29). Indeed, a recent systematic review has also recognized this limitation in the evaluation of the STS task due to variable protocols and methodological strategies even though the STS task was found to be a universal tool to track motor functional competence and musculoskeletal fitness for both clinical and research purposes (24). To the best of our knowledge, previous studies on STS measurements primarily used video recordings to evaluate postures or motion sequences and no sensor-based studies have ever been conducted in patients with PD. One recent study used non-wearable actigraphy placed under patient mattresses to determine the number of waking events and number of times patients with Alzheimer's disease left their beds (31). While a high frequency of getting out of bed was demonstrated in this study, it was not possible to capture axial and limb muscle movement patterns with non-wearable devices. To overcome this limitation and explore the feasibility of multisite wearable sensors in the measurement of the STS task, sensor evaluation was limited if the first movement of the STS task was originated by arm first, leg first, trunk first, any arm and leg together first, or trunk with any arm and/or leg first. While we appreciate that the first movement may not truly reflect the series of movement patterns of getting out of bed, we are able to identify the most common first movement pattern (trunk with any arm and/or leg first) that is shared by both PD and control subjects. Moreover, patients with PD used significantly more frequently any arm and leg together first and significantly less frequently trunk with any arm and/or leg first to perform the STS task, suggesting that patients with PD were less likely to use their axial muscles to get out of bed. This observation is consistent with a previous study with video-based analysis, demonstrating that patients with PD were significantly less likely to use a “roll off” strategy to get out of bed (12). As impaired axial rotation was identified as an underlying deficit for impaired turning in bed during the night, which also worsens as the night progresses, it is very likely that this axial impairment also exists in the early morning, explaining why patients with PD used significantly more limbs, but less trunk, as their frequent strategies to get out of bed compared to control subjects (5, 11).

Significant correlations between getting out of bed objective parameters and various clinical demographics (age, disease duration, HY staging, UPDRS axial scores, UPDRS item #28 on posture, NHQ score) suggest that there are potentially several factors contributing to the difficulties experienced by patients with PD when getting out of bed. After applying multiple linear regression analysis, age and NHQ score associated with getting out of bed objective parameters. Outside PD, the general effects of aging are also important factors that influence axial mobility and physical performance. With a direct measurement, spinal ROM and configuration (i.e. kyphosis, scoliosis) were found to worsen with aging and were also associated with various reductions in physical performances, including a supine-to-sitting task (32). Early evidence also points toward aging influences on getting out of bed strategies. For example, older women used their arms more often than young women to assist themselves to get out of bed, and even more so with arms and legs together if they experienced difficulty in this task (33). A further study also identified compensatory strategies used by older women to elevate their trunks and facilitate pivot when rising from supine to a seated position that was indicative of impaired trunk flexion (34). Viewing this limitation more simplistically, the finding that some older adults cannot sit up without hand use means that their trunk flexion ability may have declined. Additional variables that could affect a person's ability to get out of bed are many, including muscle strength, physical activity, and joint range of motion. More recently, hip muscle strength on the affected side, as shown by reduced hip adductors torque, was found to correlate with slow getting out of bed (35). Indeed, lower extremity muscle weakness was evident in patients with PD related to the loss of force production that occurs bilaterally and becomes prominent as the disease progresses (36). However, the axial function impairment and the severity, as shown by UPDRS axial scores and items related to posture, are clinically manifested by increasing rigidity of axial muscles, potentially limiting the active truncal range of motion (ROM) during getting out of bed. A recent study with isokinetic dynamometers that provided an objective measure of truncal rigidity has demonstrated a significant correlation between truncal extensor rigidity, truncal flexion, and extension ROM, as well as functional mobility in patients with PD (26). Previous studies also demonstrated a significantly reduced spinal flexibility, as measured by functional axial rotation and configuration, in patients with PD even in the early and moderate stages when the measurement of axial configurations and motions were stable and postural instability was not clinically evident (37, 38). All evidence indicates that impaired function may occur before abnormal measurements and axial symptoms are important contributors to impaired functional mobility in patients with PD and, therefore, should be evaluated early in the course of the disease even when postural instability is still asymptomatic. Further study with a larger number of participants and a comprehensive objective assessment of axial rigidity should be done to clarify the significant association between the axial rigidity and the ability to getting out of bed.

Incorporating the ability to get out of bed into a comprehensive assessment of PD has several important clinical implications that can lead to the detection of early morning symptoms that are amendable for dopaminergic medication adjustment, such as night-time long-acting dopamine agonists, control released levodopa, or dispersible levodopa formation. However, current assessment methods are limited to a few validated questionnaires and video-based analysis. Moreover, the ability to get out of bed has not been included as a primary outcome in PD clinical trials, even in studies on early morning off when getting out of bed is an essential activity that patients need to perform to start their day (21, 39). Rather, the primary outcomes of these studies still focus on the improvement of “OFF” periods, a dichotomous outcome that is based on two artificial ON-OFF states, rather than a direct assessment of a patient's daily function (40, 41). Recent advances in circuit technology have enabled researchers to apply sensors to objective monitoring of nocturnal movements in PD with significant developments made in the assessment of turning in bed ability, resulting in validated objective outcome measures (i.e. number of turns, velocity, acceleration, and angle) that have been tested in clinical trials (4, 5, 42, 43). Therefore, the application of wearable sensors could be extended to the study of getting out of bed to provide insights on pathomechanisms and provide future objective outcome measures that can be utilized in studies related to early morning off and nocturia.

Although still preliminary, this study has demonstrated the feasibility of wearable sensors in the assessment of getting out of bed in the early morning in patients with PD. Assessments were safely performed in a controlled environment and promising early objective outcome measures were identified, which should now be tested and refined in future studies. However, the limitations of this study should also be mentioned. First and foremost is the study setting, which was not the patient's own bedroom. While the current trend for assessment of night-time symptoms as primary targets is moving toward home-based monitoring, as our study is prospective, with the aim of demonstrating technological feasibility with multisite wearable sensors and patient's safety, a controlled environment was chosen. As most patients with PD are likely to be under medicated in the early morning, they are likely to be in their “OFF” state, and, thus, are at risk of losing their balance while attempting to get out of bed or during transfers from bed to chair (44, 45). Postural sway, which predisposes patients to fall, also increases when patients with PD move vertically as they get out of bed and are visually deprived (night-time and early morning) (46). In addition, testing in the patient's own bedroom also poses several confounding factors, mostly related to bedroom environments (i.e., bed height, bed sheet) that potentially influence the patient's ability to get out of bed (47, 48). Therefore, on-site research and medical staff with video monitoring were available throughout the study at the bedside to ensure safety with all subjects instructed to get out of bed at a rate they found comfortable so that they did not feel forced to perform the STS task beyond their ability. Furthermore, home-based studies are now planned to assess the feasibility of this sensor in a real-world environment. Another limitation is the objective analysis of getting out of bed patterns was restricted to the first body part, or parts, that moved. While previous video-based analysis provided important findings into the differences in movement patterns that patients with PD used to get out of bed compared to control subjects, these outcomes were mainly descriptive, making it difficult to follow longitudinally and perform statistical correlations. With multisite wearable sensors when synchronized properly, we were able to accurately identify which body parts moved during the STS task. However, as the STS task is a complex three-dimensional bed mobility action involving sequential motor skills, establishing objective movement patterns from five sensors simultaneously from the beginning to completion was exceedingly difficult due to the significant overlapping of signals from multiple sensors, making the recognition of getting out of bed activity patterns not possible. Therefore, in this study, the first body part, or parts, to move were used for analysis with confirmation of the first movements established by a set of five wearable sensors. Although preliminary, we consider these first movement patterns as valid outcomes as accuracy can be demonstrated by multisite wearable sensors and significant correlations with clinical scales have been demonstrated. Future studies should employ activity recognition processes on getting out of bed that provide diverse streams from each sensor, subsequently segmented into several time windows with specific lengths, from which feature vectors are extracted and fed to a classifier for recognition, with examples, including k-nearest neighbor, decision tree, and naïve Bayes (49, 50). One study in older people (not with PD) investigated the use of radio-frequency identification tag response to analyse bed-egress movements, proposing a bed-egress movement detection framework with a set of features derived from bed-egress motion analysis (51). In addition, future studies could incorporate biomechanical outcomes, including strength, ranges of motion, and changes in the center of pressure location to determine the ability of patients with PD to perform this task. Other limitations of our study include the small number of subjects and that the assessment of getting out of bed was limited to motor ability. Psychometric properties of getting out of bed should also be explored as it was found to be a promising tool for assessing motivation and life outlook in older adults (52).

From a patient's perspective, difficulty in getting out of bed is their most common concerning motor symptom, representing a common functional limitation that is negatively associated with the patient's quality of life (9, 19, 25, 26). From a carer's perspective, getting out of bed is the most frequent activity that patients require assistance from them to achieve and increases carer's burden (18, 53). It is also a major component of nocturnal hypokinesia that patients frequently encounter during the night, for nocturia, and early morning, as a clinical manifestation of early morning off (1). However, this clinical dimension seems to be unmatched by objective assessment methods with most current instruments video-based, supplemented by clinical rating scales.

## Conclusion

In this study, we have applied multisite wearable sensors to demonstrate the technical feasibility of this assessment approach and provided objective outcome measures of the STS task as a determinant of getting out of bed ability. Compared to control subjects, the duration of STS was significantly longer in patients with PD, associated with a significantly slower velocity of truncal rotation and a significantly greater number of any arm and leg moved together as the first movement. Importantly, these objective outcome measures significantly correlate with disease severity, especially on axial impairment, suggesting that impaired axial rotation is a contributing factor not only to difficulty turning in bed, as demonstrated in recent sensor-based studies (6, 11), but also to difficulty getting out of bed. These objective outcome measures for the ability to get out of bed should be further tested in future clinical trials to develop a battery of outcomes that are inclusive of kinematics, movement patterns, and biomechanics that can be utilized as primary objective outcome measures related to the early morning off and nocturia in patients with PD.

## Data availability statement

The raw data supporting the conclusions of this article will be made available by the authors, without undue reservation.

## Ethics statement

The studies involving human participants were reviewed and the study was approved by the Human Ethics Committee of the Faculty of Medicine, Chulalongkorn University (IRB number 153/57). All subjects gave their written informed consent prior to the enrollment of the study in accordance with the declaration of Helsinki. Written informed consent was obtained from the individual(s) for the publication of any potentially identifiable images or data included in this article.

## Author contributions

JS and RB: conception and organization. JS, CT, and RB: execution. JS: writing of the first draft. CT and RB: review and critique. All authors contributed to the article and approved the submitted version.

## Funding

This study was supported by the Senior Research Scholar Grant (RTA6280016) of the Thailand Science Research and Innovation (TSRI), the International Research Network Grant of the Thailand Research Fund (IRN59W0005), and the Center of Excellence grant of Chulalongkorn University (GCE 6100930004-1).

## Conflict of interest

Author RB has received a salary from Chulalongkorn University and a stipend from the Royal Society of Thailand; he has also received consultancy and/or honoraria/lecture fees from Abbott, Boehringer-Ingelheim, Britannia, Ipsen, Novartis, Teva-Lundbeck, Takeda, and Otsuka pharmaceuticals. He has received research funding from the Thailand Science and Research Innovation Bureau, Thailand Research Fund, Crown Property Bureau, Chulalongkorn University, and the National Science and Technology Development Agency. He holds patents for laser-guided walking stick, portable tremor device, nocturnal monitoring, and electronic Parkinson's disease symptom diary, as well as copyright on Parkinson's mascot, dopamine lyrics, and teaching video clips for common nocturnal and gastrointestinal symptoms for Parkinson's disease. The remaining authors declare that the research was conducted in the absence of any commercial or financial relationships that could be construed as a potential conflict of interest.

## Publisher's note

All claims expressed in this article are solely those of the authors and do not necessarily represent those of their affiliated organizations, or those of the publisher, the editors and the reviewers. Any product that may be evaluated in this article, or claim that may be made by its manufacturer, is not guaranteed or endorsed by the publisher.
