# Supplementary material for: Technological evaluation of strategies to get out of bed by people with Parkinson's disease: Insights from multisite wearable sensors
Source: Front Med Technol. 2022 Aug 25;4:922218. doi: 10.3389/fmedt.2022.922218 (PMC9453393; doi:10.3389/fmedt.2022.922218)
Supplement: Supplementary file 1 [file Table_1.DOCX]

Supplementary table 1: Correlation analysis between body part, or parts, that moved first and clinical characteristics of Parkinson’s disease patients

|  | **Any arm first**  **r (*p* value)** | **Any leg first**  **r (*p* value)** | **Trunk first**  **r (*p* value)** | **Any arm and leg first together**  **r (*p* value)** | **Trunk with any arm and/or leg**  **r (*p* value)** |
| --- | --- | --- | --- | --- | --- |
| Age | -0.228 (0.253) | -0.037 (0.853) | -0.066 (0.975) | 0.005 (0.998) | 0.180 (0.369) |
| Weight | -0.044 (0.827) | 0.052 (0.797) | 0.027 (0.895) | -0.220 (0.270) | 0.122 (0.546) |
| Waist circumference | -0.082 (0.686) | 0.068 (0.736) | 0.103 (0.610) | -0.246 (0.216) | 0.086 (0.670) |
| Age of onset | -0.186 (0.352) | 0.075 (0.710) | -0.038 (0.850) | -0.099 (0.624) | 0.059 (0.769) |
| Disease duration | -0.011 (0.955) | -0.209 (0.296) | 0.077 (0.704) | 0.220 (0.271) | 0.191 (0.340) |
| HY | 0.037 (0.853) | -0.126 (0.539) | 0.395 (0.041*) | 0.261 (0.189) | -0.214 (0.287) |
| UPDRS III | -0.152 (0.450) | -0.168 (0.403) | 0.281 (0.155) | 0.355 (0.069) | -0.180 (0.369) |
| UPDRS axial score | -0.210 (0.293) | -0.272 (0.170) | 0.313 (0.112) | 0.475 (0.012*) | -0.221 (0.268) |
| UPDRS item #28 (posture) | -0.252 (0.206) | -0.270 (0.173) | 0.208 (0.297) | 0.576 (0.002*) | -0.082 (0.683) |
| Total LEDD | 0.145 (0.471) | -0.022 (0.908) | -0.107 (0.596) | 0.282 (0.154) | 0.093 (0.644) |
| Nighttime LEDD | 0.214 (0.284) | -0.123 (0.516) | -0.153 (0.446) | 0.465 (0.014*) | -0.155 (0.440) |
| Total NHQ | -0.233 (0.243) | -0.152 (0.450) | 0.221 (0.269) | 0.293 (0.139) | -0.207 (0.300) |

HY: Hoehn& Yahr staging; UPDRS: Unified Parkinson’s Disease Rating Scale; LEDD: Levodopa Equivalent Dose; NHQ: Nocturnal Hypokinesia Questionnaire. Statistical significance (*) is defined when *p* ≤ 0.05.
