# Supplementary material for: Technological evaluation of strategies to get out of bed by people with Parkinson's disease: Insights from multisite wearable sensors
Source: Front Med Technol. 2022 Aug 25;4:922218. doi: 10.3389/fmedt.2022.922218 (PMC9453393; doi:10.3389/fmedt.2022.922218)

Supplementary figure 1**:** The scatter plots between age (1A), age of onset (1B), Hoehn & Yahr staging (1C), UPDRS axial score (1D), UPDSR #item 28 (1E), Nocturnal Hypokinesia Questionnaire (NHQ) score (1F) and mean duration of STS.

1A: the scatter plot between age and mean duration of STS


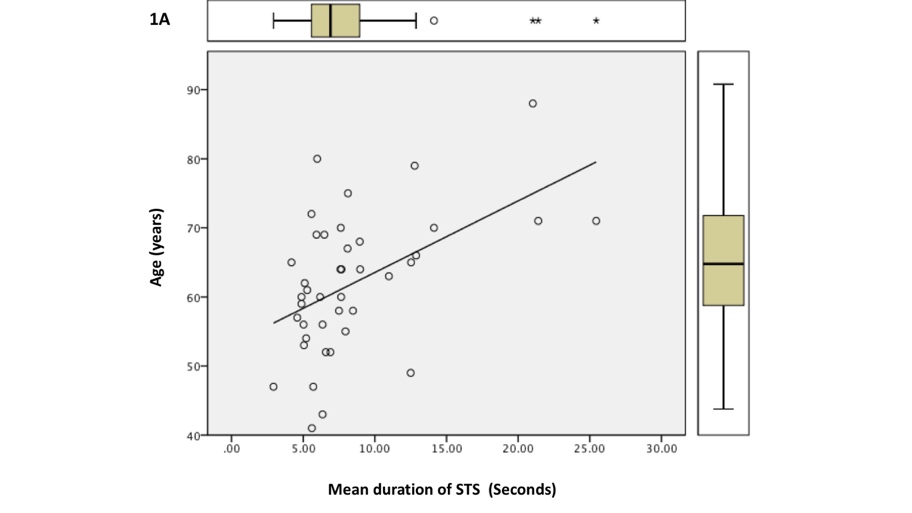


1B: the scatter plot between age of onset and mean duration of STS


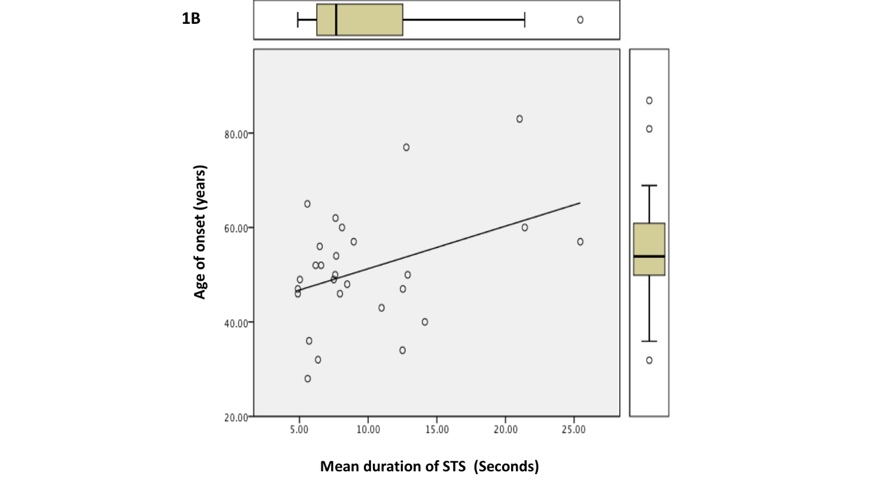


1C: the scatter plot between Hoehn & Yahr staging and mean duration of STS


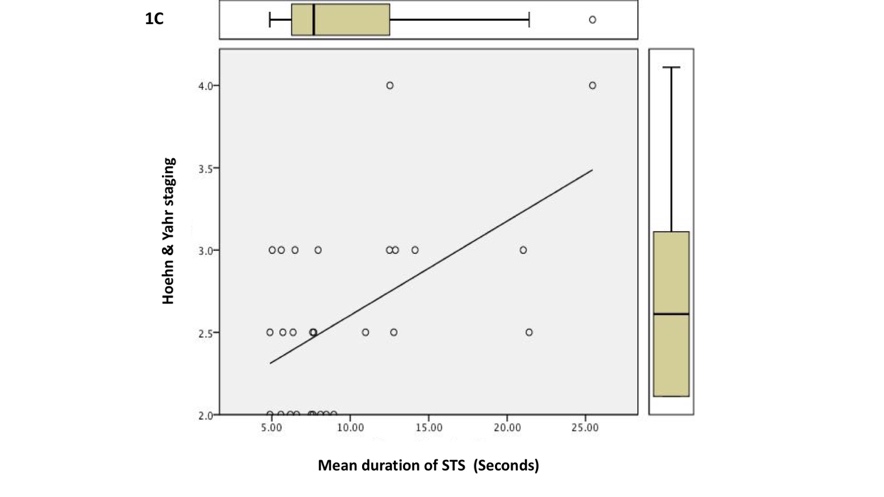


1D: the scatter plot between UPDSR axial score and mean duration of STS


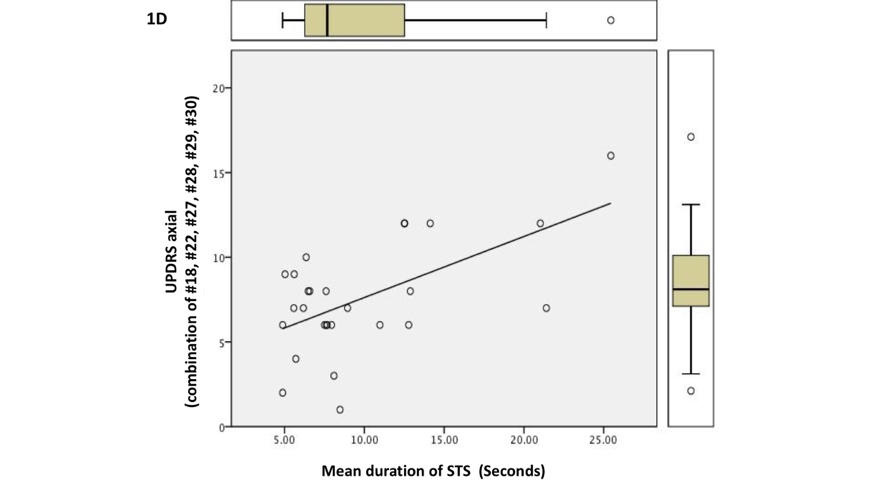


1E: the scatter plot between UPDSR #item 28 and mean duration of STS


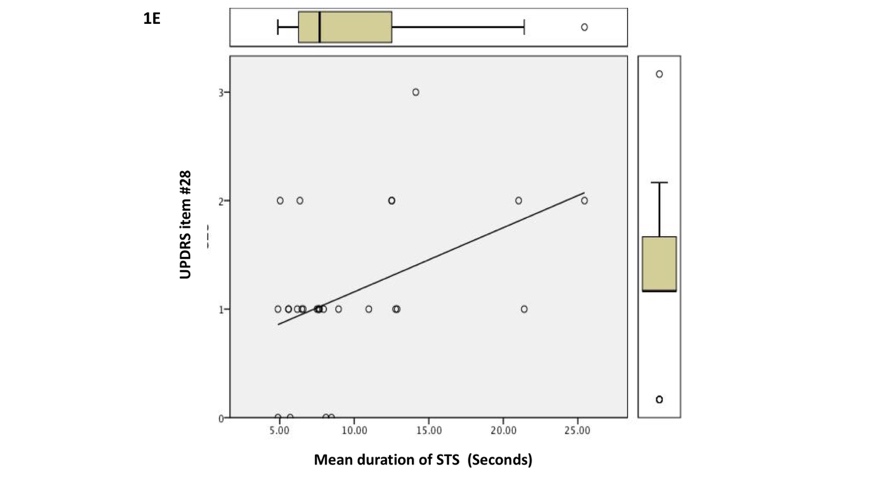


1F: the scatter plot between Nocturnal Hypokinesia Questionnaire (NHQ) score and mean duration of STS


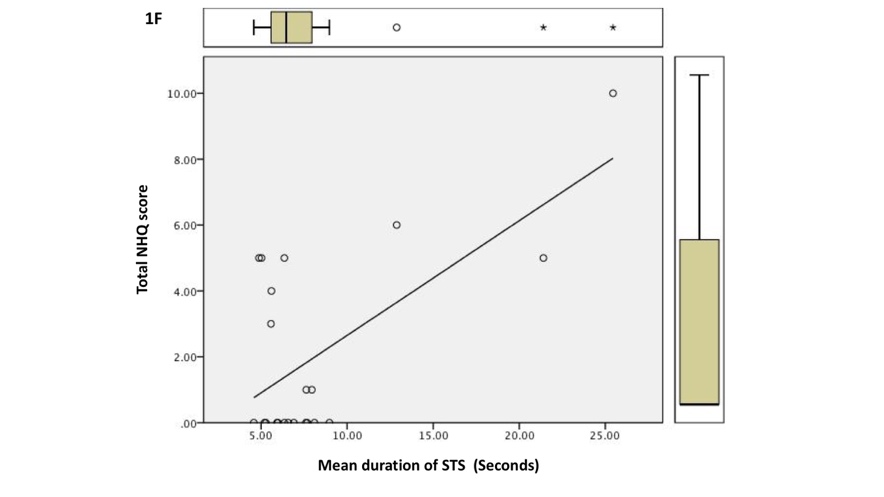


Supplementary figure 2**:** The scatter plot between Hoehn & Yahr staging (2A) and mean velocity of STS


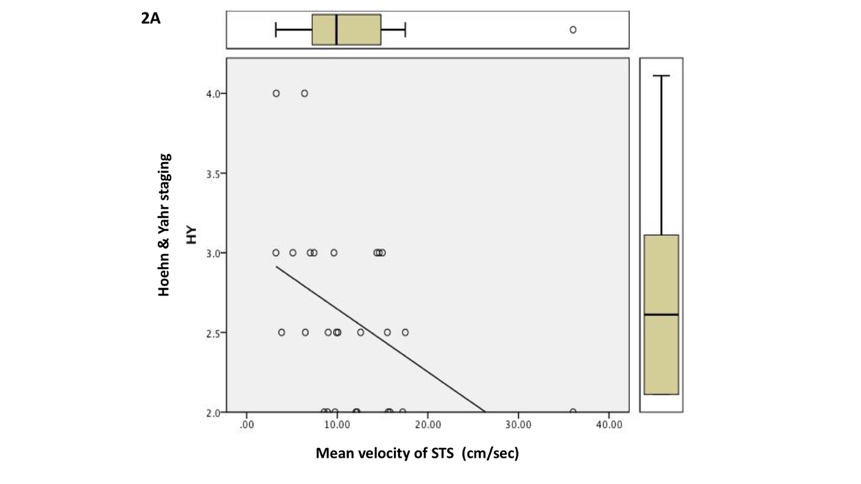

Supplement: Supplementary file 2 [file Data_Sheet_1.docx]
